# Outcomes of the Herringbone Suturing Technique for the Closure of Anterior Abdominal Wall Surgeries: A Prospective Study

**DOI:** 10.7759/cureus.107332

**Published:** 2026-04-19

**Authors:** Yamini Bhatt, Arvind Shukla, Avinash Gautam

**Affiliations:** 1 General Surgery, Mahatma Gandhi Memorial Medical College and Maharaja Yeshwantrao Hospital, Indore, IND

**Keywords:** abdominal wall closure, herringbone suturing, incisional hernia, laparotomy, ssi, wound dehiscence

## Abstract

Background

Anterior abdominal wall closure following midline laparotomy plays a crucial role in determining postoperative outcomes. Complications such as surgical site infection, wound dehiscence, and incisional hernia continue to contribute significantly to postoperative morbidity, particularly in emergency surgical settings. Conventional closure techniques may not always provide uniform tension distribution across the vertically divided linea alba. The herringbone suturing technique, characterized by a continuous crisscross pattern, has been proposed to enhance tensile strength and wound stability. This study aimed to evaluate the effectiveness and outcomes of the herringbone suturing technique for anterior abdominal wall closure following midline laparotomy.

Methodology

This prospective observational study was conducted in the Department of General Surgery at M.G.M. Medical College and M.Y. Hospital, Indore. A total of 88 patients aged 18-60 years undergoing midline laparotomy were included over a six-month period, with an additional six-month follow-up. Abdominal wall closure was performed using a single-layer continuous herringbone suturing technique with loop PDS-1 sutures. Primary outcome measures included postoperative complications such as surgical site infection, wound dehiscence, fascial dehiscence, and burst abdomen. Secondary outcomes included the incidence of incisional hernia, postoperative pain assessed using the Visual Analog Scale, length of hospital stay, and time to return to routine activities. Statistical analysis was performed using SPSS version 25, with a p-value <0.05 considered statistically significant.

Results

An uncomplicated postoperative course was observed in 78 (88.6%) patients. The overall postoperative complication rate was 11.4%. Surgical site infection was the most common complication, occurring in 10 (11.4%) patients, predominantly superficial and detected within the first 14 postoperative days. Superficial wound dehiscence occurred in seven (8.0%) patients, while fascial dehiscence was noted in three (3.4%) patients. No cases of burst abdomen were recorded. An incisional hernia was observed in one (1.1%) patient during the six-month follow-up. No significant association was found between postoperative complications and age, sex, type of surgery, or preoperative diagnosis.

Conclusions

The herringbone suturing technique appears to be a safe and effective method for anterior abdominal wall closure following midline laparotomy, with low rates of early and late postoperative complications. Its consistent performance in both emergency and elective settings suggests that it may serve as a reliable alternative to conventional closure techniques. Further multicentric studies with larger sample sizes and longer follow-up are recommended to confirm these findings.

## Introduction

The word laparos, originating from the Greek language, refers to the “soft or loose” portion of the body between the ribs and the hips, commonly describing the flanks or loins. Over the centuries, the term “laparotomy” has been widely adopted in surgical literature to describe an operative procedure involving access to and exploration of the abdominal cavity. With the advent of modern surgical practices, laparotomy has become one of the most commonly performed procedures across the globe, serving both diagnostic and therapeutic purposes in elective and emergency surgical care.

Midline laparotomy remains the most preferred incision due to its simplicity, rapidity, minimal blood loss, and excellent exposure to the peritoneal cavity and all four quadrants. However, its main drawback lies in the fact that it traverses the linea alba, an inherently weak tendinous structure. When vertically divided, the mechanical strength of the linea alba is further compromised, rendering it susceptible to postoperative complications such as wound dehiscence and incisional hernia [1,2]. Thus, while laparotomy provides unparalleled surgical access, the integrity of abdominal wall closure continues to be a cornerstone in ensuring favorable patient outcomes.

Successful closure of the abdominal wall following laparotomy is critical to promote healing, prevent postoperative morbidity, and restore abdominal wall function. Failure to achieve secure closure can result in complications such as wound dehiscence, burst abdomen, surgical site infection (SSI), and, most importantly, incisional hernia. These complications contribute to significant morbidity, prolonged hospital stay, impaired quality of life, need for secondary interventions, and increased healthcare expenditure [3,4]. Despite advances in surgical materials and techniques, postoperative incisional hernia remains a persistent problem, with reported incidences ranging from 5% to 26% in general populations [1,5] and reaching as high as 69% in certain high-risk cohorts when followed for more than three years [6]. Such figures underscore the limitations of conventional closure techniques and emphasize the need for more reliable methods that provide both immediate mechanical strength and long-term tissue stability.

Incisional hernia is one of the most frequent long-term complications following abdominal surgery. Patients may present with symptoms ranging from mild discomfort and cosmetic concerns to severe pain, bowel obstruction, or even life-threatening complications such as incarceration and strangulation [4,7]. Surgical repair of Incisional hernia, although common, can have challenges, including technical complexity, recurrence risk, and the requirement of prosthetic reinforcement in large defects. From a community perspective, incisional hernia contributes substantially to the burden on healthcare systems through increased costs, resource utilization, and loss of productivity [7].

The risk factors for incisional hernia are multifactorial. Patient-related factors such as advanced age, obesity, diabetes mellitus, malnutrition, and prior abdominal surgery play a significant role [8]. Additionally, conditions such as COPD, steroid use, and postoperative wound infection further predispose patients to poor wound healing [6]. Surgeon-related factors are equally important. The choice of suture material, technique of closure, and adherence to principles such as suture length-to-wound length ratio all directly influence outcomes [9,10]. Various strategies have been used to strengthen abdominal wall closure. Conventional interrupted sutures, while simple and reliable, are prone to uneven distribution of tension, localized ischemia, and potential for knot failure [9]. Continuous suturing methods, on the other hand, offer faster closure and uniformity but may fail if a segment of the suture line breaks or loosens [10].

Several innovative modifications have been proposed to overcome these limitations. The “small bites” technique, emphasizing a high suture-to-wound length ratio, has been shown to reduce incisional hernia by distributing tension more effectively [10]. Reinforcement techniques, such as Smead-Jones or Hughes closure, add mattress or cross-stitching components to enhance strength but may prolong operative time and increase technical complexity [10]. More recently, prophylactic mesh placement has been advocated in selected high-risk patients, but concerns regarding infection, cost, and long-term complications have limited its widespread adoption [3].

The herringbone suturing technique represents a novel modification aimed at improving abdominal wall closure. This method uses a cross-weave, interlacing stitch pattern resembling the herringbone design of textiles. The configuration ensures better tension distribution along the suture line, minimizing localized stress points and reducing the risk of tissue cut-through. Biomechanically, the herringbone stitch combines the strength of a continuous suture with the security of interrupted techniques. The knitted design allows the suture to “lock” within the tissue, preventing slippage, while simultaneously reinforcing the linea alba against intra-abdominal pressure. Early studies have suggested that this technique reduces the incidence of burst abdomen and incisional hernia, with encouraging results in terms of wound integrity and postoperative outcomes. Kothari et al. [11] first described the use of the herringbone stitch in securing abdominal wall closure during emergency laparotomy, reporting favorable outcomes with reduced complication rates compared to conventional interrupted sutures. Similarly, Shukla et al. [12] conducted a comparative study demonstrating the superiority of this technique in terms of wound security, postoperative morbidity, and long-term outcomes. These findings indicate that the herringbone stitch may represent a simple, cost-effective, and reproducible alternative to conventional closure techniques, particularly in resource-limited settings [13,14].

Given the high burden of incisional hernia [15] and wound complications [16,17] in clinical practice, particularly in emergency surgeries, research data are necessary to validate the safety, efficacy, and reproducibility of this technique. The present study was designed as a prospective evaluation of the outcomes of the herringbone suturing technique for anterior abdominal wall closure in patients undergoing laparotomy [18-20].

## Materials and methods

Study setting and duration

This study was conducted in the Department of General Surgery at MGM Medical College and MY Hospital, Indore, a tertiary care teaching institution catering to a large urban and rural population. The study duration was six months from the date of ethical committee approval (approval number: IRB00007879; dated 09/11/24), with an additional follow-up period of six months for each recruited patient from November 2024 to October 2025.

Study design

The study was designed as a prospective observational study to evaluate the effectiveness of the herringbone suturing technique for anterior abdominal wall closure in patients undergoing laparotomy.

Study population and sample size

The study population comprised patients admitted to the surgical ward who underwent laparotomy through a midline incision. The sample size was calculated based on a previous study by Kothari et al. (2018) [11], which reported favorable outcomes with herringbone closure in emergency laparotomy. Using the findings of this study, a total sample size of 88 patients was determined to be adequate for the present study.

Patients aged between 18 and 60 years who underwent laparotomy through a midline incision and provided informed written consent were included in the study. Patients younger than 18 years or older than 60 years were excluded. Additional exclusion criteria included patients with a serum albumin level of less than 3.5 g/dL at the time of surgery and those who were unwilling to give consent to participate in the study.

All enrolled patients underwent a detailed preoperative assessment. This included the collection of demographic details such as age, sex, occupation, and place of residence. Clinical data, including presenting complaints, associated comorbidities, and nutritional status, were documented. Relevant laboratory and imaging investigations were reviewed as part of the diagnostic workup. Details regarding management, including preoperative optimization measures, were also recorded. All collected data were systematically documented using a predesigned proforma.

Surgical procedure

**Figure 1:**
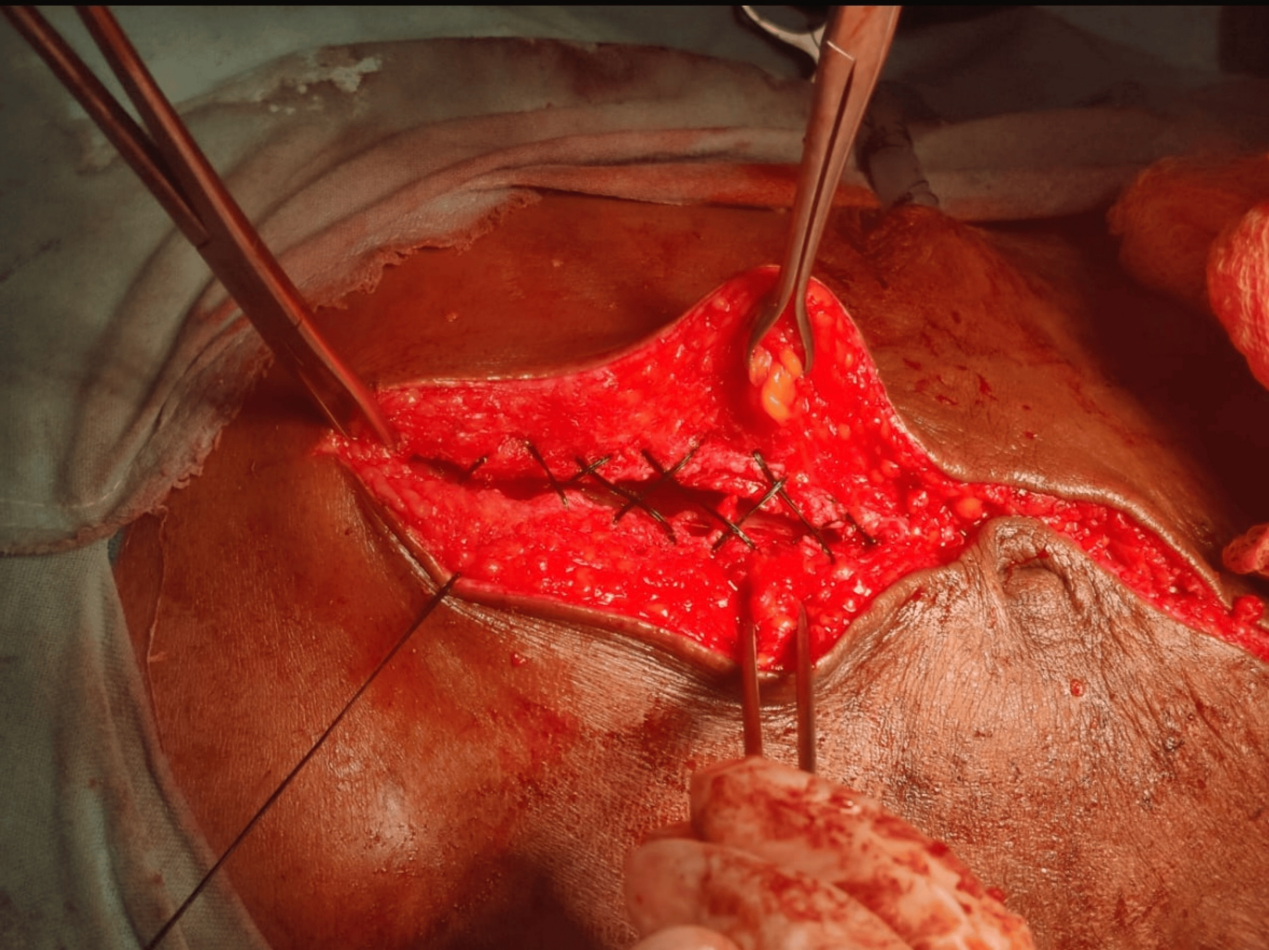
Herringbone suturing technique.

After standard preoperative preparation and induction of anesthesia, a midline laparotomy incision was made. Following completion of the primary surgical procedure, the abdominal wall was closed using the herringbone suturing technique (Figure 1). The closure was performed in a single-layer continuous crisscross pattern, inspired by an embroidery-style lattice arrangement, with the aim of distributing tensile forces both longitudinally and transversely along the incision line.

**Figure 2:**
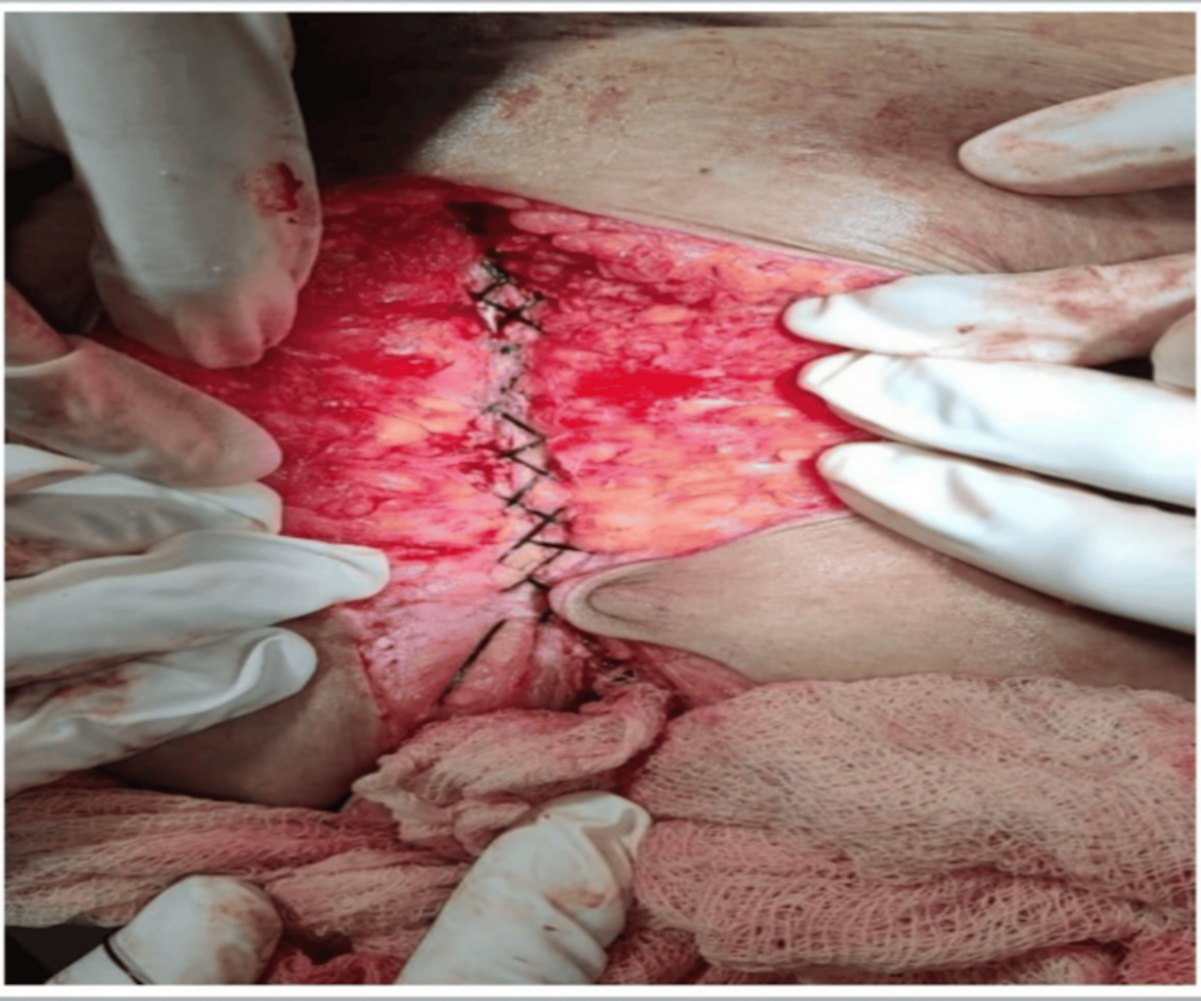
Intraoperative image.

In all patients, a loop polydioxanone (PDS) suture was used for abdominal wall closure. “Far” bites were taken approximately 2 cm from the wound edge, while “near” bites were placed about 1 cm from the wound edge, close to the linea alba (Figure 2). Knots were buried within the rectus sheath to reduce the risk of stitch granuloma formation. Throughout the procedure, meticulous care was taken to avoid incorporation of visceral structures and to ensure that excessive tension was not applied to the suture line.

Postoperative care and follow-up

Postoperatively, all patients were managed according to the standard institutional protocols. This included regular monitoring of vital signs and systematic assessment of postoperative pain using the Visual Analog Scale (VAS) at predetermined intervals. Surgical wounds were inspected routinely for evidence of complications such as SSI, seroma, hematoma, or wound dehiscence. Early ambulation was encouraged in all patients, and nutritional optimization was ensured as part of the postoperative care plan.

Patients were followed up at 14 days, three months, and six months after surgery. At each follow-up visit, wound healing status, postoperative complications, and patient-reported outcomes were assessed and documented in a structured manner.

The primary outcome measures of the study included the postoperative complication rate, encompassing SSIs, wound dehiscence, fascial dehiscence, and burst abdomen, as well as the length of hospital stay and mortality rate. Secondary outcome measures included the incidence of incisional hernia within six months of surgery, patient-reported pain and discomfort assessed using the VAS ranging from 0 to 10, time taken to return to routine daily activities, and the need for reoperation.

SSI was defined as an infection occurring within 30 days of surgery, or within one year in cases involving implants, and was classified into superficial, deep, and organ/space SSI. Superficial SSI involved the skin and subcutaneous tissue, deep SSI involved the fascial and muscle layers, and organ/space SSI involved organs or spaces accessed during the surgical procedure. Wound dehiscence was defined as partial or complete separation of the approximated wound edges occurring within five to eight days postoperatively. A burst abdomen was described as a sudden separation of the musculo-aponeurotic layers of the abdominal wall, often presenting with bowel protrusion. An incisional hernia was defined as a type of ventral hernia occurring at the site of a previous laparotomy incision and was typically diagnosed clinically during the follow-up period.

Statistical analysis

Data collected in the study were entered and analyzed using SPSS version 25 (IBM Corp., Armonk, NY, USA). Continuous variables, such as age, were summarized using mean ± standard deviation (SD) and median with interquartile range (IQR), while categorical variables, including sex, type of laparotomy, preoperative diagnosis, and postoperative complications, were expressed as frequencies and percentages. Associations between categorical variables and postoperative outcomes were assessed using chi-square tests or Fisher’s exact tests where appropriate. A p-value <0.05 was considered statistically significant.

## Results

Age distribution in the study population

Table 1 shows the age-wise distribution of patients included in the study. The majority of patients belonged to the ≤30-year age group (32 out of 88 (36.4%)), followed by those aged >50 years (19 out of 88 (21.6%)). The mean age of the study population was 38.26 ± 16.55 years, indicating a wide age variability. The median age was 35.0 years with an IQR of 23.75-48.5 years.

**Table 1:** Age distribution of the study population. Values are presented as frequency (%), mean ± standard deviation (SD), and median with interquartile range (IQR). No inferential statistical test was applied, as this table describes the baseline characteristics of the study population.

Age group (years)	Number (n)	Percentage (%)	Mean ± SD (years)	Median (IQR) (years)
≤30	32	36.4	23.6 ± 3.6	23 (21–26)
31–40	21	23.9	35.6 ± 2.9	35 (33–38)
41–50	16	18.2	45.0 ± 3.1	45 (42–48)
>50	19	21.6	65.0 ± 6.8	65 (60–70)
Total	88	100	38.26 ± 16.55	35.0 (23.75–48.5)

Sex distribution in the study population

Table 2 depicts the sex distribution of the study population. A marked male predominance was observed, with males constituting 69 out of 88 (78.4%), while females comprised 19 (21.6%). This male preponderance could be attributed to the higher incidence of trauma, perforation, peritonitis, and emergency abdominal conditions among males. Similar trends have been reported in previous surgical studies involving emergency laparotomies. The skewed sex distribution highlighted the demographic pattern of patients presenting for abdominal surgeries in the study setting.

**Table 2:** Sex distribution of the study population. A goodness-of-fit chi-square test demonstrated that the proportion of males was significantly higher than that of females in the study population (χ² = 28.41, p < 0.001). P-value <0.05 is considered significant.

Sex	Number (n)	Percentage (%)	Chi-square value	P-value
Male	69	78.4	28.41	<0.001*
Female	19	21.6		
Total	88	100		

Preoperative diagnosis distribution in the study population

Table 3 illustrates the distribution of patients according to preoperative diagnosis. Perforation peritonitis was the most common indication for laparotomy (28 (31.8%)). This was followed by subacute intestinal obstruction (11 (12.5%)), blunt trauma to the abdomen (10 (11.4%)), and penetrating abdominal injuries (9 (10.2%)).

**Table 3:** Disease distribution. Values are expressed as frequency and percentage. No inferential statistical test was applied, as this table describes the distribution of preoperative diagnoses in the study population.

Preoperative diagnosis	Number (n)	Percentage (%)
Perforation peritonitis	28	31.8
Subacute intestinal obstruction	11	12.5
Blunt trauma abdomen	10	11.4
Penetrating/Stab injury to the abdomen	9	10.2
Corrosive/Unknown substance ingestion	5	5.7
Abdominal lump	3	3.4
Koch’s abdomen	3	3.4
Appendicitis (acute)	2	2.3
Rectal pathology (perforation/prolapse/sigmoidectomy)	2	2.3
Pseudocyst pancreas	2	2.3
Ruptured liver abscess (±bowel perforation)	2	2.3
Malignancy (colon, esophagus, adenocarcinoma)	5	5.7
Hernia (obstructed/incarcerated inguinal)	2	2.3
Sigmoid volvulus	1	1.1
Gastric outlet obstruction	1	1.1
Polytrauma with abdominal injury	1	1.1
Diaphragmatic hernia with bladder rupture	1	1.1
Total	88	100

Type of laparotomy distribution in the study population

Table 4 shows the distribution of patients based on the type of laparotomy performed. A large majority of patients underwent emergency laparotomy (71 (80.7%)), while only 17 (19.3%) patients underwent elective procedures. This predominance of emergency surgeries correlated with the high incidence of perforation peritonitis and traumatic abdominal injuries observed in the study.

**Table 4:** Emergency versus elective surgeries. A goodness-of-fit chi-square test demonstrated that emergency laparotomy was performed significantly more frequently than elective laparotomy in the study population (χ² = 33.14, p < 0.001). P-value <0.05 is considered significant.

Type of laparotomy	Number (n)	Percentage (%)	Chi-square value	P-value
Emergency	71	80.7	33.14	<0.001*
Elective	17	19.3		
Total	88	100		

Postoperative complication distribution in the study population

Table 5 shows the distribution of individual postoperative complications following the herringbone suturing technique. SSI was observed in 10 (11.4%) patients, making it the most common complication. Superficial wound dehiscence occurred in seven (8.0%) patients, while fascial dehiscence was noted in only three (3.4%) patients. Incisional hernia was observed in a single patient (1.1%) during follow-up. The chi-square test showed that the absence of postoperative complications was significantly higher compared with their occurrence (χ² = 26.87, p < 0.001).

**Table 5:** Types of postoperative complications. The chi-square test showed that the absence of postoperative complications was significantly higher compared with their occurrence (χ² = 26.87, p < 0.001). P-value <0.05 is considered significant.

Complication	Present, n (%)	Absent, n (%)	Chi-square value	P-value
Surgical site infection	10 (11.4)	78 (88.6)	26.87	<0.001*
Superficial wound dehiscence	7 (8.0)	81 (92.0)		
Fascial dehiscence	3 (3.4)	85 (96.6)		
Incisional hernia	1 (1.1)	87 (98.9)		

Onset of postoperative complications during follow-up

Table 6 illustrates the timing of the onset of postoperative complications during follow-up. All 10 cases of SSI were observed within the first 14 postoperative days. Superficial (six cases) and fascial (two cases) wound dehiscence predominantly occurred in the early postoperative period, with very few cases (one case) presenting at three months. No new cases of wound dehiscence were observed at six months. A single case of incisional hernia was detected at the sixth-month follow-up, indicating its delayed presentation. The chi-square test demonstrated that most postoperative complications occurred within the first 14 postoperative days (χ² = 18.42, p = 0.001).

**Table 6:** Onset of complications. The chi-square test demonstrated that most postoperative complications occurred within the first 14 postoperative days (χ² = 18.42, p = 0.001). P-value <0.05 is considered significant.

Complication	14 days, n (%)	3 months, n (%)	6 months, n (%)	Chi-square value	P-value
Surgical site infection	10 (11.4)	0	0	18.42	0.001*
Superficial wound dehiscence	6 (6.8)	1 (1.1)	0		
Fascial dehiscence	2 (2.3)	1 (1.1)	0		
Incisional hernia	0	0	1 (1.1)		

Postoperative outcome

Table 7 presents the overall postoperative outcome using a composite complication analysis. The majority of patients (78 out of 88 (88.6%)) did not develop any postoperative complications. Only 10 out of 88 (11.4%) patients experienced one or more postoperative complications during the follow-up period.

**Table 7:** Postoperative outcomes. P-value <0.05 is considered significant.

Outcome	Number (n)	Percentage (%)	Chi-square value	P-value
No postoperative complication	78	88.6	52.55	<0.001*
≥1 postoperative complication	10	11.4		
Total	88	100		

Association between the type of laparotomy and overall postoperative complications

Table 8 analyzed the association between the type of laparotomy and overall postoperative complications. Complications were observed in nine (12.7%) patients undergoing emergency laparotomy compared to one (5.9%) patient in elective cases. However, this difference was not statistically significant (p = 0.62). This indicated that the type of surgery did not significantly influence the occurrence of postoperative complications. The findings suggested that the herringbone suturing technique performed consistently in both emergency and elective settings.

**Table 8:** Type of surgery and postoperative complications. The difference was not statistically significant on the chi-square test (χ² = 0.63, p = 0.62). P-value <0.05 is considered significant.

Type of surgery	Complication present, n (%)	No complication, n (%)	Total	P-value
Emergency (n = 71)	9 (12.7)	62 (87.3)	71	0.62
Elective (n = 17)	1 (5.9)	16 (94.1)	17	
Total	10	78	88	
Chi-square	0.63		-	-

Type of surgery versus surgical site infection

Table 9 shows the relationship between the type of surgery and SSI. SSI was more commonly observed in emergency surgeries in nine (12.7%) patients compared to one (5.9%) patient in elective surgeries. However, the difference was not statistically significant (p = 0.48). This suggested that the occurrence of SSI was not significantly influenced by whether the surgery was performed as an emergency or elective procedure.

**Table 9:** Surgical site infections (SSIs) in types of laparotomy. The difference was not statistically significant on the chi-square test analysis (χ² = 0.63, p = 0.48). P-value <0.05 is considered significant.

Type of surgery	SSI present, n (%)	SSI absent, n (%)	P-value
Emergency	9 (12.7)	62 (87.3)	0.48
Elective	1 (5.9)	16 (94.1)	
Chi-square	0.63		-

Age group versus overall postoperative complications

Table 10 depicts the association between age groups and overall postoperative complications. Complication rates were relatively comparable across all age groups. Although a slightly higher complication rate was observed in patients aged above 50 years, the difference was not statistically significant (p = 0.84).

**Table 10:** Age group versus postoperative complications. Statistical analysis using the chi-square test showed no significant association between age group and postoperative complications (χ² = 0.59, p = 0.84). P-value <0.05 is considered significant.

Age group (years)	Complication present, n (%)	No complication, n (%)	Total	P-value
≤30 (n = 32)	3 (9.4)	29 (90.6)	32	0.84
31–40 (n = 21)	2 (9.5)	19 (90.5)	21	
41–50 (n = 16)	2 (12.5)	14 (87.5)	16	
>50 (n = 19)	3 (15.8)	16 (84.2)	19	
Total	10	78	88	
Chi-square	0.59		-	-

Sex versus overall postoperative complications

Table 11 demonstrates the association between sex and overall postoperative complications. Complications were observed in 8 out of 69 (11.6%) male patients and 2 out of 19 (10.5%) female patients. The difference was not statistically significant (p = 0.91). This suggested that sex did not influence the risk of postoperative complications. The herringbone suturing technique showed similar outcomes in both male and female patients.

**Table 11:** Sex versus overall postoperative complications. No statistically significant association was noted on the chi-square test (χ² = 0.017, p = 0.91). P-value <0.05 is considered significant.

Sex	Complication present, n (%)	No complication, n (%)	Total	P-value
Male (n = 69)	8 (11.6)	61 (88.4)	69	0.91
Female (n = 19)	2 (10.5)	17 (89.5)	19	
Total	10	78	88	
Chi-square	0.017		-	-

Postoperative pain assessment using the Visual Analog Scale

Table 12 shows that postoperative pain scores significantly declined over time. Analysis using repeated-measures analysis of variance showed a statistically significant reduction in mean VAS scores from postoperative day 1 to day 14 (F = 152.4, p < 0.001).

**Table 12:** Postoperative pain assessment using the Visual Analog Scale (VAS). Statistical test: repeated-measures analysis of variance. P-value <0.05 is considered significant. P-value <0.001 is statistically significant, implying that the patients had less postoperative pain following the herringbone suturing technique.

Postoperative day	Mean VAS score ± SD	Median (IQR)	Interpretation	Test statistic	P-value
Day 1	6.8 ± 1.2	7 (6–8)	Moderate to severe pain	F = 152.4	<0.001*
Day 3	4.5 ± 1.1	4 (4–5)	Moderate pain		
Day 7	2.3 ± 0.9	2 (2–3)	Mild pain		
Day 14	0.8 ± 0.6	1 (0–1)	Minimal pain		

Preoperative diagnosis versus overall postoperative complications

Table 13 compares the overall postoperative complications between patients with perforation peritonitis and other diagnoses. Overall, 5 out of 28 (17.9%) patients with perforation peritonitis showed a higher complication rate compared to those with other diagnoses in 5 out of 60 (8.3%) patients. However, this difference was not statistically significant (p = 0.29). This indicated that preoperative diagnosis did not significantly influence postoperative outcomes. The technique remained effective even in high-risk pathological conditions.

**Table 13:** Preoperative diagnosis versus overall postoperative complications. No statistically significant association was noted on the chi-square test (χ² = 1.72, p = 0.29). P-value <0.05 is considered significant.

Diagnosis group	Complication present, n (%)	No complication, n (%)	P-value
Perforation peritonitis (n = 28)	5 (17.9)	23 (82.1)	0.29
Other diagnoses (n = 60)	5 (8.3)	55 (91.7)	
Total	10	78	
Chi-square	1.72		-

## Discussion

Midline laparotomy remains one of the most commonly used abdominal incisions, yet it is associated with a substantial risk of postoperative complications, particularly incisional hernia, with reported rates ranging from 5% to 20% [1,2,5,6]. Incisional hernias significantly impair quality of life, body image, and functional outcomes, while also imposing a considerable economic burden due to the need for complex surgical repair [4,7]. Both patient-related factors, such as malnutrition, obesity, smoking, and comorbid illness, and operative factors, including contamination and closure technique, contribute to impaired wound healing and fascial failure [1,2].

The technique of abdominal wall closure plays a pivotal role in reducing these complications. Continuous fascial closure using slowly absorbable monofilament sutures with an adequate suture-to-wound length ratio has consistently demonstrated lower rates of wound dehiscence and incisional hernia compared with interrupted techniques [8,9,14,15]. However, outcomes remain variable, particularly in emergency laparotomies, where tissue edema, contamination, and increased intra-abdominal pressure predispose to closure failure.

In this study of patients undergoing midline laparotomy, a clear male predominance was observed, which was statistically significant on the chi-square test (χ² = 28.41, p < 0.001). This may reflect greater exposure of males to risk factors such as trauma, perforation, peritonitis, and other emergency surgical conditions. The religious distribution largely mirrored the regional demographic pattern and was also statistically significant (χ² = 69.14, p < 0.001).

Emergency laparotomy constituted the majority of cases (80.7%), significantly exceeding elective procedures (χ² = 33.14, p < 0.001). This predominance is expected in tertiary care settings where acute abdominal emergencies form a major surgical workload.

Postoperative complications included SSI, superficial wound dehiscence, fascial dehiscence, and incisional hernia. SSI was the most frequent complication, consistent with previously published literature. Most complications occurred within the first 14 postoperative days, which was statistically significant on the chi-square test (χ² = 18.42, p = 0.001).

Although complications were more frequent following emergency procedures compared with elective surgery, this difference was not statistically significant (p = 0.62). Similarly, no significant association was observed between postoperative complications and patient age or sex. These findings suggest that while emergency surgeries constitute the majority of cases, demographic factors alone may not significantly influence complication rates.

The herringbone suturing technique represents a modification of continuous closure designed to counter multidirectional mechanical forces acting on midline incisions. By distributing tensile stress both longitudinally and transversely, this lattice-like configuration may reduce focal strain at the linea alba. Prior studies by Kothari et al. and Shukla et al. have reported favorable outcomes with this technique in emergency settings, including reduced wound complications and improved fascial integrity [11,12]. While mesh reinforcement has shown benefit in selected elective cases, its routine use in contaminated fields remains controversial [3]. Therefore, optimized suture-based techniques such as the herringbone method may offer a practical and safe alternative. Larger randomized trials with long-term follow-up are required to confirm its role in preventing incisional hernia.

Midline laparotomy is associated with significant early and late postoperative complications, notably wound dehiscence, burst abdomen, SSI, and incisional hernia. Wound dehiscence occurs in approximately 0.4-3.5% of cases but may reach 10% in emergency and resource-limited settings, with reported mortality as high as 45% [1,2]. Burst abdomen, a severe form of fascial dehiscence, typically presents between postoperative days 7 and 10 and carries mortality rates of 25-45%, particularly following emergency surgery, peritonitis, or wound infection [1,2,8]. Consistently identified risk factors include hypoalbuminemia, anemia, diabetes, obesity, chronic lung disease, ascites, wound infection, and emergency laparotomy [1,2,5,6].

Incisional hernia represents the most common long-term complication, with rates ranging from 9% to 20% at one year and increasing progressively with longer follow-up, reaching up to 30-40% in high-risk groups [1,5,6,10]. Emergency surgery, contamination, poor closure technique, and surgeon-related factors significantly influence hernia development [1,9,15]. SSIs further compound morbidity, with laparotomy-associated SSI rates reported between 17% and 23%, especially in contaminated and prolonged procedures [1].

Evidence strongly supports continuous mass closure using slowly absorbable monofilament sutures and an adequate suture-to-wound length ratio to reduce complications [9,14,15]. Novel techniques such as small-bite closure, far-near-near-far repairs, and lattice-based patterns, such as the herringbone stitch, show promise in redistributing tension and reducing dehiscence and hernia rates, particularly in emergency settings [11-13,15]. However, high-quality randomized trials with long-term follow-up remain necessary to establish definitive superiority.

Studies supporting herringbone suturing in laparotomy closure

Kothari et al. (2018) [11] conducted a prospective observational study in central India to assess the effectiveness of the herringbone stitch for abdominal wall closure in emergency midline laparotomy. A total of 220 patients were included, with 112 undergoing herringbone closure and 108 receiving standard continuous closure using polypropylene sutures. Demographic characteristics were comparable across groups. The study found that while the rates of SSI, knot granuloma, and superficial wound dehiscence were similar, the herringbone group had significantly lower fascial dehiscence and incisional hernia rates. The authors highlighted that the herringbone stitch distributes tension parallel to the wound, making it technically simple, reproducible, and effective. They concluded that this technique offers superiority over conventional rectus sheath closure in emergency laparotomy.

Shukla et al. (2024) [12] conducted a comparative study at MGM Medical College and MY Hospital, Indore, evaluating the herringbone stitch versus interrupted suturing for rectus closure in emergency laparotomy. In total, 50 patients were included, with 25 patients in each group. Both groups showed a similar incidence of SSI; however, superficial wound dehiscence, fascial dehiscence, and incisional hernia were lower in the herringbone group. The findings suggest that the herringbone technique offers superior strength and reduced postoperative complications compared to the interrupted method. This highlights its potential as a preferred closure technique in emergency settings.

Fortelny et al. (2022) [13] reviewed closure techniques for elective midline laparotomy, focusing on outcomes without mesh reinforcement. Literature evidence indicates that incisional hernia occurs in 5-20% of such cases, making technique selection critical. The study compared long stitch and short stitch methods, highlighting parameters such as suture-to-wound length ratio, stitch number, and spacing. Findings showed that continuous small-bite closure with slowly absorbable sutures and a suture-to-wound ratio of ≥5:1 significantly reduced wound complications and incisional hernia compared to large-bite closure. The study concluded that the small-bite technique represents the best available method for elective midline closure.

Van den Berg et al. (2025) [14] performed a meta-analysis of 41 randomized controlled trials and 9 cohort studies assessing suture techniques for laparotomy closure. They found no major differences between interrupted and continuous suturing or between slow- and fast-absorbable sutures. However, the small-bite technique with slowly absorbable sutures significantly reduced incisional hernia, fascial dehiscence, and SSI. Modified Smead-Jones and retention-line sutures showed promise in emergency cases. No superiority was noted for layered or Hughes closure over mass closure. The study concluded that small-bite, slowly absorbable sutures are optimal for elective laparotomy closure.

Stephens et al. (2025) [15] performed an international cross-sectional survey on abdominal wall closure practices in emergency laparotomy across 32 countries. Responses from 234 surgeons revealed that 85.8% used small-bite closure, but only 42.2% followed the standard 5 mm spacing. Measurement of the suture-to-wound ratio was rare (7.7%). Loop PDS was the preferred suture (42.7%), while self-locking and antiseptic-coated sutures were used infrequently. Prophylactic mesh augmentation was adopted by just 10%, mainly in the retrorectus space. The study concluded that uptake of modern closure techniques remains inconsistent in emergency surgery.

Maheshwari et al. (2024) [16] reported a comprehensive review of advances in subcuticular suturing for abdominal wall closure. The study discussed the evolution of this technique, emphasizing its benefits in terms of cosmetic outcomes, reduced infection rates, and improved patient satisfaction. Traditional continuous and interrupted methods were compared with newer approaches such as barbed and knotless sutures. Evidence from clinical trials and case studies highlighted better healing efficacy, fewer complications, and cost-effectiveness with modern subcuticular techniques. Despite certain challenges, the review concluded that subcuticular suturing holds significant promise for enhancing surgical outcomes. The study stressed the importance of ongoing research and innovation in this field.

Garg et al. (2023) [17] conducted a prospective observational study comparing the far-near-near-far technique with conventional rectus sheath closure in emergency exploratory laparotomy. The study included patients with a mean age of 42.14 years, and both groups were comparable in terms of demographics and comorbidities. Operative and closure times were not significantly different between the techniques. Postoperative complications such as wound infection, dehiscence, burst abdomen, sinus formation, and incisional hernia were also similar. Late complications showed no significant variation. The study concluded that the far-near-near-far method offers outcomes comparable to conventional closure, thus providing surgeons with flexibility in technique selection.

Chatterjee et al. (2021) [18] conducted a prospective randomized controlled study at Bankura Sammilani Medical College, West Bengal, to compare the impact of short stitch and long stitch techniques on midline laparotomy closure. A total of 104 patients were enrolled, with 51 in the short stitch group and 53 in the long stitch group. The analysis showed significantly fewer complications in the short stitch group, with lower rates of SSI (11.7% vs. 24.5%), wound dehiscence (5.8% vs. 15%), and incisional hernia (7.8% vs. 20.7%). All differences were statistically significant. The authors concluded that short stitch closure reduces postoperative morbidity and is superior to the long stitch technique for midline laparotomy wounds.

Agrawal et al. (2019) [19] conducted a prospective study at SBRKM Government Medical College, Jagdalpur, to evaluate the outcomes of single-layer midline abdominal wall closure. Over a 24-month period, 52 patients underwent closure with non-absorbable continuous sutures, with separate skin closure. The mean patient age was 46.5 years, with a male-to-female ratio of 3:1; 35 underwent emergency, and 17 underwent elective laparotomies. The study reported wound infection in 38.4% and wound gaping in 19.2% of cases, but no incisional hernia was observed within six months of follow-up. The authors concluded that single-layer closure is fast, cost-effective, and provides adequate strength, with complication rates comparable to existing randomized controlled trials and meta-analyses.

Gandhi et al. (2016) [20] conducted a retrospective study at Seth G.S. Medical College and KEM Hospital, Mumbai, to evaluate abdominal wall closure techniques in emergency laparotomies. The study included 126 patients with indications such as inflammatory, traumatic, and neoplastic conditions, all managed through vertical midline incisions. The closure technique used showed wound infection in 9.52% of patients, while only 2.38% developed wound dehiscence (burst abdomen). These results indicated a lower incidence of postoperative complications compared to conventional expectations. The authors concluded that their method of abdominal closure was effective in reducing burst abdomen, thereby minimizing morbidity, mortality, and healthcare costs.

Berretta et al. (2010) [21] conducted a randomized prospective study involving 191 patients with gynecological cancers to assess abdominal wall closure techniques after median laparotomy. Patients were allocated into three groups, namely, en bloc closure of peritoneum and fascia with Premilene, en bloc closure with polydioxanone, and separate closure using Ethibond single stitches. Statistical analysis showed no significant differences among groups regarding incisional hernia, wound dehiscence, infection, or scar pain. The overall incidence of incisional hernia was 8%. The authors concluded that no suture type or closure method demonstrated clear superiority. They emphasized that minimizing abdominal wall trauma with minimally invasive techniques may be the best preventive approach.

Hollinsky et al.(2006) [22] investigated the tensile strength of scar tissue compared with intact ventral abdominal wall structures using post-mortem samples from 66 individuals. Tissue from the linea alba, anterior and posterior rectus sheath, and scar tissue following laparotomy was subjected to tensile loading. Results demonstrated that scar tissue had significantly reduced strength compared with normal abdominal wall tissue in both horizontal and vertical directions. For example, in the epigastric region, horizontal load tolerance was 10.0 N/mm² for linea alba versus 6.9 N/mm² for scar tissue, and vertical load was 4.5 versus 3.3 N/mm², respectively. Similar reductions were observed in the hypogastric region. The authors concluded that scar tissue’s reduced load-bearing capacity contributes to a permanent risk of incisional hernia, emphasizing the importance of secure closure and potential reinforcement strategies.

Richards et al. (1983) [23] conducted a randomized prospective trial including 571 patients to compare continuous and interrupted techniques for abdominal wound closure. Patients were stratified based on wound type, i.e., clean, clean-contaminated, or contaminated. In midline incisions, dehiscence occurred in 2.0% of the continuous group versus 0.9% of the interrupted group, while ventral hernia developed in 2.0% and 0.5%, respectively; these differences were not statistically significant. For oblique incisions, dehiscence and hernia rates were negligible in both groups. The study emphasized that surgical technique quality influenced outcomes more than the closure method. Continuous suturing required nearly half the operative time compared to interrupted suturing, leading the authors to recommend continuous closure for its efficiency and comparable safety.

Limitations

This study was conducted at a single center with a relatively small sample size and lacked a control group using conventional closure techniques. The follow-up duration of six months may be insufficient to assess long-term complications such as incisional hernia. Additionally, the technique requires greater operative time and may involve a learning curve for surgeons. No American Society of Anesthesiologists grading was performed, and emergency and elective laparotomies were generalized.

## Conclusions

This prospective observational study evaluated the outcomes of the herringbone suturing technique for anterior abdominal wall closure following midline laparotomy. The technique demonstrated favourable outcomes, with most patients experiencing an uncomplicated postoperative course and an overall complication rate of 11.4%. SSI was the most common complication, followed by superficial wound dehiscence and fascial dehiscence. An incisional hernia occurred in only one patient during the six-month follow-up period. Most complications were observed within the first 14 postoperative days. Statistical analysis using the chi-square test showed no significant association between postoperative complications and age, sex, type of laparotomy, or underlying diagnosis. Despite a predominance of emergency surgeries, the herringbone suturing technique maintained low rates of wound failure, suggesting effective tension distribution and secure fascial closure. Postoperative pain assessed using the VAS showed a gradual reduction from postoperative day 1 to day 14, indicating good patient tolerance. Overall, the herringbone suturing technique appears to be a safe and effective alternative for midline laparotomy closure. Larger multicentric studies with longer follow-up are recommended to further validate these findings.

## References

[1] Bosanquet DC, Ansell J, Abdelrahman T (2015). Systematic review and meta-regression of factors affecting midline incisional hernia rates: analysis of 14,618 patients. PLoS One.

[2] Höer J, Lawong G, Klinge U, Schumpelick V (2002). [Factors influencing the development of incisional hernia. A retrospective study of 2,983 laparotomy patients over a period of 10 years]. Chirurg.

[3] Jairam AP, Timmermans L, Eker HH (2017). Prevention of incisional hernia with prophylactic onlay and sublay mesh reinforcement versus primary suture only in midline laparotomies (PRIMA): 2-year follow-up of a multicentre, double-blind, randomised controlled trial. Lancet.

[4] van Ramshorst GH, Eker HH, Hop WC, Jeekel J, Lange JF (2012). Impact of incisional hernia on health-related quality of life and body image: a prospective cohort study. Am J Surg.

[5] Fink C, Baumann P, Wente MN (2014). Incisional hernia rate 3 years after midline laparotomy. Br J Surg.

[6] Itatsu K, Yokoyama Y, Sugawara G (2014). Incidence of and risk factors for incisional hernia after abdominal surgery. Br J Surg.

[7] Gillion JF, Sanders D, Miserez M, Muysoms F (2016). The economic burden of incisional ventral hernia repair: a multicentric cost analysis. Hernia.

[8] Niggebrugge AH, Trimbos JB, Hermans J, Steup WH, Van De Velde CJ (1999). Influence of abdominal-wound closure technique on complications after surgery: a randomised study. Lancet.

[9] Mudge M, Hughes LE (1985). Incisional hernia: a 10 year prospective study of incidence and attitudes. Br J Surg.

[10] (2022). Incisional hernia following colorectal cancer surgery according to suture technique: Hughes Abdominal Repair Randomized Trial (HART). Br J Surg.

[11] Kothari R, Thakur R, Sharma D, Agarwal P (2018). Herring bone stitch: knitting to secure abdominal wall closure for emergency midline laparotomy. Surg Med Open Acc J.

[12] Shukla AK, Gautam A, Shrivastava S, Arora S, Singh R, Athya RK (2024). Herringbone stitch technique versus interrupted suturing technique for rectus closure in emergency laparotomy: a comparative study. Asian J Med Sci.

[13] Fortelny RH (2018). Abdominal wall closure in elective midline laparotomy: the current recommendations. Front Surg.

[14] van den Berg R, Visscher L, Menon AG, Deerenberg EB, Tanis PJ (2025). Suture techniques and materials for fascial closure of abdominal wall incisions: a comprehensive meta-analysis. Ann Surg Open.

[15] Stephens IJ, Kelly E, Ferreira F, Boermeester MA, Sugrue ME (2025). Variable use of modern abdominal wall closure techniques at emergency laparotomy - an international, cross-sectional survey of surgical practice. Eur J Trauma Emerg Surg.

[16] Maheshwari M, Khan IA (2024). Advances and techniques in subcuticular suturing for abdominal wall closure: a comprehensive review. Cureus.

[17] Garg S, Yadav MS, Singhal K (2023). A clinical comparative study of rectus sheath closure techniques in emergency exploratory laparotomy: evaluating "far-near-near-far" vs. conventional closure approach. Cureus.

[18] Chatterjee S, Bhattacharya T (2021). A study to evaluate the effects of various abdominal closure techniques on midline laparotomy wounds in a tertiary care hospital in West Bengal. Int Surg J.

[19] Agrawal SN, Singh K (2019). A prospective study of single layer abdominal wall closure in the tertiary care hospital. Int Surg J.

[20] Gandhi JA, Shinde PH, Digarse RD (2016). Evaluation of abdominal wall closure technique in emergency laparotomies at a tertiary care hospital. Int Surg J.

[21] Berretta R, Rolla M, Patrelli TS (2010). Randomised prospective study of abdominal wall closure in patients with gynaecological cancer. Aust N Z J Obstet Gynaecol.

[22] Hollinsky C, Sandberg S (2007). Measurement of the tensile strength of the ventral abdominal wall in comparison with scar tissue. Clin Biomech (Bristol).

[23] Richards PC, Balch CM, Aldrete JS (1983). Abdominal wound closure. A randomized prospective study of 571 patients comparing continuous vs. interrupted suture techniques. Ann Surg.

